# To what extent do post-traumatic mental health and other problems reflect pre-existing problems? Findings from the prospective comparative population-based VICTIMS-study

**DOI:** 10.1177/00207640221140287

**Published:** 2022-12-04

**Authors:** Peter G. van der Velden, Carlo Contino, Marcel Das, Lutz Wittmann

**Affiliations:** 1 Centerdata, Tilburg, The Netherlands; 2 TRANZO, Tilburg School of Social and Behavioral Sciences, Tilburg University, Tilburg, The Netherlands; 3 Fonds Slachtofferhulp, Den Haag, The Netherlands; 4 Tilburg School of Economics and Management, Tilburg University, Tilburg, The Netherlands; 5 International Psychoanalytic University Berlin, Berlin, Germany

**Keywords:** PTSD, anxiety, depression, financial, legal, prospective

## Abstract

**Background:**

Findings from prospective studies question the assumption that mental health problems observed in traumatized adults mainly reflect the effects of potentially traumatic events.

**Aims:**

Aim of the present comparative prospective study is to clarify the extent to which victims of potentially traumatic events with mental health, social, financial, and/or legal problems, already suffered from such problems before these events.

**Method:**

Data was extracted from three surveys of the prospective VICTIMS-study (T1 = 2018, T2 = 2019, T3 = 2020), conducted with the population-based longitudinal LISS-panel. Differences between victims (*n* = 340, victimized by violence, accidents, and serious threats in the 12 months before T3) and nonvictims (*n* = 3,872, not victimized by such events in this period), were examined using multivariate logistic regression analyses.

**Results:**

The large majority of victims with current (at T3) anxiety and depression symptomatology (74%), general mental health problems (71%), partner/family (67%), financial (76%), and legal problems (58%), and lack of support (79%), already had these problems (at T1 and/or at T2). A similar pattern was observed among nonvictims. Of the victims with current probable PTSD (at T3), 87% already had any mental health problem. At T3, among both groups, the incidence of problems was substantially lower than their prevalence. The large majority of victims with post-event mental health, social, financial, and legal problems already suffered from these problems in the past.

**Conclusions:**

When victims seek help for their problems, professional care providers should be aware that in most cases, as among nonvictims, these problems are chronic/re-current rather than new problems.

## Introduction

Mental health problems in the aftermath of potentially traumatic events (PTEs), such as symptoms of posttraumatic stress, anxiety, and depression, are well documented. Epidemiological studies among civilian populations have shown that after most types of PTEs, a minority of victims meet the criteria of a mental disorder such as posttraumatic stress disorder or major depression (Alisic et al., 2014; Koenen et al., 2017; Vibhakar et al., 2019). Other problems reported by victims of PTEs include somatic symptoms, work-related problems, lack of social support, as well as legal and financial problems (Eberhard-Gran et al., 2007; Jordan, 2004; Loya, 2015; Magnavita et al., 2019; Mayou et al., 2002; O’Donnell et al., 2005; Schatman & Thoman, 2015; van der Velden et al., 2019). They all may interact such as that post-trauma financial problems may cause stress and thereby intensify posttraumatic stress symptoms (Galea et al., 2008; Kiely et al., 2015; van der Velden et al., 2019; van der Velden, Contino et al., 2020).

However, in a systematic review of prospective studies on risk factors for PTSD mostly among high-risk populations (soldiers and rescue workers), DiGangi et al. (2013) concluded that many post-trauma variables such as psychopathology and social-ecological factors were actually present *before* the index trauma. Similarly, Danese et al. (2017) found that impairments in cognitive functions (such as memory, perceptual reasoning, and verbal comprehension) *predated* childhood victimization. A review by Scheeringa (2021) on the association of neurobiological differences with PTSD found that 19 of the identified 25 studies testing the diathesis stress theory had positive results, for example, found that neurobiological differences existed prior to exposure. These findings suggest that PTSD symptomatology manifests mainly in individuals in which psychopathology and social-ecological factors were actually present before the index trauma. Importantly, many (non-trauma) longitudinal studies show that mental health problems are predominantly and strongly predicted by (similar) mental health problems assessed at earlier stages (Altmann & Roth, 2020; Bosmans et al., 2019; Cuijpers & Smit, 2004; Jacobson & Newman, 2017; Kaniasty & Norris, 2008; Oe et al., 2016; van der Velden et al., 2016).

Based on such evidence, one may expect that part of the victims with post-event mental health problems already suffered from similar mental health problems before the PTEs, suggesting that the incidence of (new) post-trauma mental health problems is (much) lower than their prevalence. The extent to which victims with other post-trauma problems, such as financial problems, legal problems, and lack of social support already had these problems in the years before being victimized is – to the best of our knowledge – unknown.

Insight into pre-existing problems among those with post-trauma problems may help us to improve our understanding of the determinants of post-trauma mental health, social, financial, and legal problems. This in turn may enable us to optimize the efficacy of clinical interventions as well as of the cooperation between different involved service providers (Shorey et al., 2014).

However, current post-trauma conducted studies among civilians are not equipped to examine this topic due to the absence of prospective pre-trauma assessments (Brewin, 2007; Dekel & Bonanno, 2013; Hyman IE & Loftus, 1998; Southwick et al., 1997). Almost all studies on the effects of PTEs were conducted after these events. Furthermore, available prospective trauma-studies (compare DiGangi et al., 2013) have largely focused on mental health outcomes only, and included mostly high-risk or other specific populations, limiting the representativeness of reported findings to the general population. In addition, it is unknown if nonvictims with similar current problems compared to victims differ in the prevalence of pre-existing problems.

### The current study

To gain more insight into the role of pre-existing problems, a three-wave prospective comparative population-based study was conducted to answer the following research questions:

(1) What is the prevalence of mental health problems (anxiety and depression symptoms, posttraumatic stress symptoms, general mental health problems), social problems (lack of emotional support, problems at work, problems with partner/family), financial, and legal problems prior to PTEs (i.e. pre-existing problems) among victims of recent PTEs compared to nonvictims in the same period?
(2) What is the prevalence of current (post-trauma) problems among victims of recent PTEs compared to nonvictims?
(3) What is the prevalence of pre-existing problems among those victims of recent PTEs with current problems compared to nonvictims?
(4) What is the incidence of new (post-trauma) problems among victims of recent PTEs compared to nonvictims?

To answer these research questions, the prospective VICTIMS-study was designed.

## Methods

### Participants and procedure

The Victims in Modern Society (VICTIMS) study is conducted using the Dutch Longitudinal Internet studies for the Social Sciences (LISS) panel (Scherpenzeel & Das, 2011). The set-up of this panel is funded by the Dutch Research Council (NWO) and administered by Centerdata. The LISS panel is based on a large traditional probability sample drawn from the Dutch population register by Statistics Netherlands (CBS). Importantly, respondents who do not have a computer and/or internet access are provided with the necessary equipment at home. Panel members receive an incentive of €15 per hour for their participation. For further information about open-access data LISS-panel see https://www.dataarchive.lissdata.nl/(in English). In accordance with the General Data Protection Regulation (GDPR), participants gave explicit digital consent for the use of the collected data for scientific and policy relevant research.

Surveys were conducted in March 2018 (T1, *N*^invited^ = 7,292, response = 82.1%), March 2019 (T2, *N*^invited^ = 6,298, response = 83.2%), and March 2020 (T3, *N*^invited^ = 6,568, response = 83.6%). Reminders were sent a month later. The questionnaire of the surveys was approved by an Internal Review Board, consisting of a panel of internal and external reviewers of Centerdata not involved in the design of the VICTIMS-study. The authors assert that all procedures contributing to this work comply with the ethical standards of the relevant national and institutional committees on human experimentation and with the Helsinki Declaration of 1975, as revised in 2008.

In total, 4,237 panel members participated in all three surveys. To optimize the representativeness of the study sample, we used the following strategy. We first composed 32 exclusive demographic profiles, based on sex (male, female), age (18–34, 35–49, 50–64, 65 years and older), marital status (married, unmarried), and employment status (employed, unemployed) among the total adult Dutch population based on the data of Statistics Netherlands. These 32 profiles were used to weight the study sample (based on variables collected at T1). All results are based on the total weighted sample. Twenty-six victims were excluded as they filled in event-related questionnaires with an event in mind which did not meet event-related inclusion criteria.

### Measures

In all three surveys, the following questionnaires were administered besides the assessment of sociodemographic characteristics.

#### Potentially traumatic events and stressful life events

Exposure to potentially traumatic events (PTEs) and major stressful life events (SLEs) in the past 12 months was examined by means of a list of 21 events derived from existing questionnaires on PTEs and SLEs (Bronner et al., 2009; de Vries & Olff, 2009; Hentschel et al., 2017; van der Velden et al., 2013). Since we focused on the 12-month prevalence, events like adverse childhood experiences and WWII were eliminated. The following items examined exposure to PTEs: (1) *physical violence*: sexual violence/sexual abuse (not online); online sexual violence/sexual abuse; robbery; physical violence, but not by own partner; physical violence by own partner; (2) *accidents*: traffic accidents, disasters, fire, medical errors; and (3) *serious threats*: serious threats; without the use of physical violence (not online); online serious threats; without use of physical violence.

Further items covered experiences of SLEs such as the death of a (significant) other, serious infections (HIV, AIDS), and physical diseases (cancer, heart attack). Respondents were asked to indicate for all 21 events, if they experienced a specific event (1 = no, 2 = yes) in the 12 months before the survey. Additionally, participants were also given the opportunity to report events in the previous 12 months that were not listed, which were recoded afterward into existing or new categories. Victims were asked to rate the level of stress during the event (1 = not or barely to 5 = very much).

#### Anxiety and depression symptomatology

Anxiety and depression symptomatology was assessed using the Mental Health Inventory (MHI-5; Means-Christensen et al., 2005; Ware JE & Sherbourne, 1992). The MHI-5 asks respondents to rate their mental health during the past month on 6-point Likert scales (0 = never to 5 = continuously). After recoding the negatively formulated items, the total scores were computed and multiplied by four (to arrive at a 0–100 scale), where lower scores indicate higher anxiety and depression symptom levels (all Cronbach’s α’s ⩾ .87). Cut-off scores of 60 (score < 60) and 45 (score < 45) were used to identify respondents with moderate-severe and severe anxiety depression symptomatology (abbreviated as ADS), respectively (Driessen, 2011).

#### Probable PTSD

*P*robable PTSD was assessed using the 8-item version of the PTSD Checklist for DSM-5 (PCL-5; Pereira-Lima et al., 2019; Price et al., 2016; van der Velden et al., 2018; Weathers, 2008) which examines symptoms across the four symptom clusters of PTSD according to DSM-5 (APA, 2013). The PCL-5 items focus on symptoms in the past month and have 5-point Likert scales (0 = not at all to 4 = extremely). A cut-off of 13 was applied to identify victims with probable PTSD (Pereira-Lima et al., 2019). When respondents reported two or more events, they were asked to focus on the most impactful or stressful PTE when filling in the PCL-5 (see Appendix 1; all Cronbach’s α’s ⩾ .93).

#### General mental health problems to legal problems

To examine general mental health problems (abbreviated as GMHP), problems at work, problems in the family/with partner, financial problems, and legal problems, the brief screening Problems and Help Inventarisation-List (PHIL; van der Velden & Kleber, 2018; van der Velden et al., 2019) was administered in all surveys. The single items of the PHIL assess the presence of problems in the aforementioned areas (1 = yes, 2 = no).

#### Lack of emotional support

Lack of emotional support in response to problems was examined using the eight-item subscale Lack of emotional support of the Social Support List-Discrepancy (SSL-D; Bridges et al., 2002; van Sonderen, 2012). The SSL-D items apply 4-point Likert scales (1 = I miss this, I would like it to happen more often to 4 = It happens too often). For the present study, total scores were subtracted from the total maximum scores. Higher scores reflect more lack of emotional support (all Cronbach’sα’s > .89). A cut-off of ⩾12 was used to identify respondents with high lack of emotional support (van der Velden, Contino et al., 2020).

### Statistical Analyses

For research question 1 (Prevalence of pre-existing problems): differences between victims and nonvictims in the prevalence of pre-existing problems were assessed using multivariate logistic regression analyses with demographics (sex and age at T1, education level, employment status, marital status at T1 and T2), and stressful life events (in the past 12 months before T1 and T2) as control variables.

For research question 2 (Prevalence of current problems): similar logistic regression analyses were used to assess differences in the prevalence of current problems between both groups, using demographics at T3 and stressful life events in the past 12 months before T3 as control variables.

For research question 3 (Prevalence of pre-existing problems among those with current problems): identical analyses as the previous ones were conducted to assess differences in the prevalence of pre-existing problems among those with current problems between both groups. These analyses were then repeated in order to quantify the prevalence of *any* rather than only the *same* pre-existing mental health problem (anxiety and depression, probable PTSD, general mental health problems) and *any* pre-existing social/financial/legal problem (at T1 and/or T2) among those with current problems.

For research question 4) (incidence of new problems): the incidence of (new) problems was defined by having problems at T3 while not having problems at T1 and T2, and similar analyses as the previous ones were conducted to assess differences in the incidence of (new) problems between both groups.

For all control variables, a stepwise procedure (*p*-value IN = .05; *p*-value OUT = 0.10) was applied to limit the number of control variables by excluding variables that were not significantly associated with the dependent variable.

### Non-response analyses

Of the 5,879 respondents at T1, 4,237 (72.1%) participated at T2 and T3. Non-response analyses using multivariate logistic regression analyses with non-response as the dependent variable (1 = participated at T1 vs. 2 = participated at T1, T2, and T3) revealed the following differences. Married respondents participated more often than unmarried respondents (adjusted OR [aOR] = 1.30, 95% confidence interval (95% CI) = 1.15 to 1.47, *p* < .001), and respondents of ⩾35 years old more often than 18-34 years old respondents (aOR^35–49^ = 1.32, 95% CI = 1.11–1.57, *p* = .002; aOR^50–64^= 2.24, 95% CI = 1.87–2.68, *p* < .001; aOR^65+^ = 2.17, 95% CI = 1.78 to 2.63, *p* < .001). Responders less often had moderate-severe anxiety and depression symptoms at T1 (15.3%) than non-responders (20.5%; aOR = 0.81, 95% CI = 0.68–0.97, *p* = .020), but not less often severe anxiety and depression symptom at T1 (*p* = .713). No differences in other demographics and problems were found. Importantly, the study sample was weighted for employment status, gender, marital status, and age. Due to the weighting, the prevalence of moderate-severe anxiety and depression symptoms among the weighted study sample (16.6%) was comparable to the prevalence among all respondents at T1 (16.8%).

## Results

### Characteristics of samples

Table 1 shows that at T3, victims (*n* = 340) were significantly younger and less often married, and more often confronted with potentially traumatic events in the 12 months before T1 and T2, and other stressful life-events in the 12 months before T1, T2, and T3 than nonvictims (*n* = 3871). Of the victims, 67.1% reported that they experienced rather-very much stress during the event (not shown in Table 1). For an overview of experiences with VAT-events, we refer to Appendix 1.

**Table 1.** Characteristics of study samples.

	Nonvictims	Victims	χ^2^	*p*
	(*N* = 3,871)	(*N* = 340)		
	*n* (%)	*n* (%)		
Sex				
Male	1,895 (49.0)	177 (52.1)	1.20	.272
Female	1976 (51.0)	163 (47.9)		
Age				
18–34 years	873 (22.6)	95 (27.9)	17.21	<.001
35–49 years	891 (23.0)	89 (26.2)		
50–64 years	996 (25.7)	93 (27.4)		
65 years	1,111 (28.7)	63 (18.5)		
Education^a^				
Low	954 (24.6)	75 (22.1)	1.14	.567
Medium	1,359 (35.1)	124 (36.5)		
High	1,558 (40.2)	141 (41.5)		
Married				
Yes	1,954 (50.5)	210 (61.8)	15.98	<.001
No	1,918 (49.6)	130 (38.2)		
Primary occupation				
Employed	1781 (46.0)	160 (46.9)	0.01	.749
Not Employed	2,089 (54.0)	181 (53.2)		
VAT in 12 months before T1				
No	3,440 (88.9)	247 (72.6)	75.46	<.001
Yes	431 (11.1)	93 (27.4)		
VAT in 12 months before T2				
No	3,366 (87.0)	230 (67.4)	133.43	<.001
Yes	374 (9.7)	111 (32.6)		
Other stressful life-events in 12 months before T1				
No	2,497 (64.5)	188 (55.1)	11.91	<.001
Yes	1,374 (35.5)	153 (44.9)		
Other stressful life-events in 12 months before T2				
No	2562 (66.2)	192 (56.5)	13.03	<.001
Yes	1,309 (33.8)	148 (43.5)		
Other stressful life-events in 12 months before T3				
No	2637 (68.1)	181 (53.2)	31.29	<.001
Yes	1,234 (31.9)	159 (46.8)		

a Low = primary school, intermediate secondary education, US: junior high school; Medium = higher secondary education/preparatory university education, US: senior high school, intermediate vocational education, US: junior college; High = higher vocational education, US: college, university, according to education level categories of Statistics Netherlands (CBS). All results are based on the weighted sample. Due to weighting numbers may slightly differ.

T1 = March 2018, T2 = March 2019, and T3 = March 2020.

### Prevalence of pre-existing problems

Table 2 shows the prevalence of pre-existing problems and results of the comparisons. Victims compared to nonvictims had a significantly higher prevalence of all assessed *pre-existing* problems.

**Table 2.** Prevalence of pre-existing and current problems among victims and nonvictims.

	*N* ^total^	Prevalence pre-existing problems^a^		Prevalence current problems^b^	
		*n* (%)	aOR (95% CI)	*n* (%)	aOR (95% CI)
Moderate-severe anxiety and depression symptoms					
Nonvictims	3,871	853 (22.0)	1	605 (15.6)	1
Victims	340	121 (35.6)	1.64 (1.28–2.10)***	95 (27.9)	1.75 (1.35–2.28)***
Probable PTSD (VAT-related)^c^					
Nonvictims	3,872	110 (2.8)	1	–	
Victims	340	43 (12.6)	3.74 (2.53–5.52)***	67 (19.7)	n.a.
General mental health problems					
Nonvictims	3,871	560 (14.5)	1	397 (10.3)	1
Victims	340	87 (25.6)	1.64 (1.25–2.15)***	75 (22.1)	1.91 (1.42-2.57)***
Problems at work^d^					
Nonvictims	2,023	283 (14.0)	1	141 (7.0)	1
Victims	187	41 (21.9)	1.76 (1.21–2.55)**	26 (13.9)	1.70 (1.17-2.48)**
Problems with partner/family					
Nonvictims	3,871	439 (11.3)	1	264 (6.8)	1
Victims	340	78 (22.9)	2.17 (1.64–2.86)***	46 (13.5)	1.92 (1.37–2.71)***
Financial problems					
Nonvictims	3,871	371 (9.6)	1	227 (5.9)	1
Victims	340	86 (25.3)	2.71 (2.04–3.59)***	59 (17.4)	2.69 (1.94–3.73)***
Legal problems					
Nonvictims	3,872	120 (3.1)	1	53 (1.4)	1
Victims	340	27 (7.9)	2.37 (1.53–3.68)***	19 (5.6)	3.67 (2.13–6.32)***
Lack of emotional support					
Nonvictims	3,871	1,448 (37.4)	1	896 (23.1)	1
Victims	340	171 (50.3)	1.56 (1.24–1.96)***	120 (35.3)	1.70 (1.34–2.16)***

*Note*. Due to weighting numbers may differ slightly. N.a. = not applicable; 95% CI = 95% confidence interval of aOR.

a Problems at T1 and/or T2 [Percentage = (*n*^prevalence before T3^/*N*^total^) × 100]. aOR = Odds ratio adjusted for sex, age, employment status, marital status and/or education level at T1 and T2, and/or other PTEs/SLEs in the 12 months before T1 and /or T2.

b Problems at T3 [Percentage = (*n*^prevalence T3^/*N*^total^) × 100].]. aOR = Odds ratio adjusted for sex, age, employment status, marital status and/or education level at T3, and/or other PTEs/SLEs in the 12 months before T3.

c By group definition, nonvictims do not suffer from VAT-related probable PTSD (VAT in 12 months before T3) at T3.

d Among those employed at T3.

T1 = March 2018, T2 = March 2019, and T3 = March 2020.

* *p* < .05, **p < .01, ***p < .001.

### Prevalence of current problems

Table 2 additionally shows the prevalence of current problems and results of the comparisons. According to the adjusted Odd ratios, victims compared to nonvictims had a significantly higher prevalence of all assessed *current* problems.

### Prevalence of pre-existing problems among those with current problems

As shown in Table 3, many respondents of both groups who suffer from current problems already had the same pre-existing problems. For example, of the victims with current general mental health problems, 70.7% had pre-existing general mental health problems compared to 73.3% of the nonvictims.

**Table 3.** Prevalence of pre-existing problems among victims and nonvictims with current problems and incidence of problems.

	*N* ^total^	Prevalence problems before T3 among those with problems at T3^a^		Incidence problems at T3^b^	
		*n* (%)	aOR (95% CI)	*n* (%)	aOR (95% CI)
Moderate-severe anxiety and depression symptoms					
Nonvictims	605/3,871	409 (67.6)	1	196 (5.1)	1
Victims	95/340	70 (73.7)	1.22 (0.74-2.02)	25 (7.4)	1.51 (0.98-2.32)
Probable PTSD (VAT-related)^c^					
Nonvictims					
Victims	67/340	32 (47.8)	n.a.	32 (9.4)	n.a.
General mental health problems (GMHP)					
Nonvictims	397/3,871	291 (73.3)	1	106 (2.7)	1
Victims	75/340	53 (70.7)	0.77 (0.43-1.36)	22 (6.5)	2.10 (1.29-3.41)**
Problems at work^d^					
Nonvictims	141,2023	66 (46.8)	1	75 (3.7)	1
Victims	26/187	14 (53.8)	1.36 (0.59-3.13)	12 (6.4)	1.69 (0.90-3.18)
Problems with partner/family					
Nonvictims	264/3,871	148 (56.1)	1	116 (3.0)	1
Victims	46/340	31 (67.4)	1.66 (0.85-3.23)	16 (4.7)	1.55 (0.90-2.67)
Financial problems					
Nonvictims	227/3,871	147 (64.8)	1	80 (2.1)	1
Victims	59/340	45 (76.3)	1.75 (0.88-3.49)	14 (4.1)	1.62 (0.90-2.91)
Legal problems					
Nonvictims	53/3,872	21 (39.6)		31 (0.8)	
Victims	19/340	11 (57.9)	n.c.	8 (2.4)	n.c.
Lack of emotional support					
Nonvictims	896/3,871	652 (72.8)	1	245 (6.3)	1
Victims	120/340	95 (79.2)	1.41 (0.88-2.27)	25 (7.4)	1.16 (0.75-1.78)

a Problems at T1 and/or T2 among those problems at T3 [percentage = (*n*^prevalence before T3 among T3^/*n*^prevalence T3^) × 100.aOR = Odds ratio adjusted for sex, age, employment status, marital status and/or education level at T3, and/or other PTEs/SLEs in the 12 months before T3.

b Problems at T3, but not at T1 and not at T2 [percentage^incidence^ = (*n*^incidence^/*N*^total^) × 100]. aOR = Odds ratio adjusted for sex, age, employment status, marital status and/or education level at T3, and/or other PTEs/SLEs in the 12 months before T3.

c By group definition, nonvictims do not suffer from VAT-related probable PTSD (VAT in 12 months before T3) atT3.

d Among those employed at T3.

T1 = March 2018, T2 = March 2019, and T3 = March 2020.

Due to weighting numbers may differ slightly. N.a. = not applicable. n.c. = not computed because of low cell counts of denominator. aOR = Odds ratio adjusted for sex, age, employment status, marital status and/or education level at T3, and/or other PTEs/SLEs in the 12 months before T3. 95% CI = 95% confidence interval of aOR.

* *p* < .05, **p < .01, ***p < .001.

The prevalence of any of the assessed pre-existing problems among victims and nonvictims with specific current problems are presented in Table 4. Results of the comparisons show that pre-existing mental health problems (ADS, GMHP, and/or probable PTSD) were present in the large majority of victims with current anxiety and depression symptoms (80.0%) and general mental health problems (79.7%) as well as of nonvictims (71.4% and 83.4%, respectively). Both groups did not differ significantly on this issue. The same pattern was found for pre-existing social, legal, and financial problems which were present in the majority of victims and nonvictims with current mental health problems. Only for anxiety and depression symptoms, a significant difference emerged: victims with current moderate-severe anxiety and depression symptoms more often had any of the assessed pre-existing mental health problems than nonvictims.

**Table 4.** Prevalence of any pre-existing mental health and any other problem among victims and nonvictims with problems at T3.

	Prevalence of any pre-existing mental health problem^a^ among those with current problems			Prevalence of any pre-existing SocFinLeg problem^b^ among those with current problems	
	*N* ^total^	*n* (%)	aOR (95% CI)	*n* (%)	aOR (95% CI)
Moderate-severe a Anxiety and depression symptoms					
Nonvictims	605	432 (71.4)	1	460 (76.0)	1
Victims	95	76 (80.0)	1.34 (0.77–2.32)	83 (87.4)	2.15 (1.13–4.09)*
Probable PTSD (VAT-related)					
Nonvictims					
Victims	67	58 (86.6)	n.a.	58 (86.6)	n.a.
General mental health problems					
Nonvictims	397	331 (83.4)	1	314 (79.1)	1
Victims	74	59 (79.7)	0.70 (0.37–1.36)	64 (86.5)	1.58 (0.78–3.19)
Problems at work^c^					
Nonvictims	141	68 (48.2)	1	112 (79.4)	1
Victims	26	13 (50.0)	1.03 (0.45–2.36)	22 (81.5)	1.39 (0.45–4.27)
Problems with partner/family					
Nonvictims	264	135 (51.1)	1	222 (84.1)	1
Victims	46	34 (73.9)	1.97 (0.92–4.22)	39 (84.8)	1.02 (0.41–2.52)
Financial problems					
Nonvictims	227	119 (52.4)	1	199 (87.7)	1
Victims	59	38 (64.4)	1.02 (0.52–2.00)	57 (96.6)	4.37 (0.85–22.48)
Legal problems					
Nonvictims	52	23 (44.2)		44 (84.6)	1
Victims	19	10 (52.6)	1.02 (0.24–4.26)	18 (94.7)	5.38 (0.41–70.83)
Lack of emotional support					
Nonvictims	896	414 (46.2)	1	715 (79.8)	1
Victims	120	80 (66.7)	2.00 (1.32–3.04)**	105 (87.5)	1.69 (0.95–2.99)

a Moderate-severe anxiety and depression symptoms (ADS), probable PTSD, and/or general mental health problems (GMHP) at T1 and/or T2.

b Social, financial, and/or legal problems (SocFinLeg problems) at T1 and/or at T2.

c Among those employed at T3.

Due to weighting numbers may differ slightly. T1 = March 2018, T2 = March 2019, and T3 = March 2020. n.a. = not applicable. n.c. = not computed because of low cell counts of denominator. aOR = Odds ratio adjusted for sex, age, employment status, marital status and/or education level at T3, and/or other PTEs/SLEs in the 12 months before T3. 95% CI = 95% confidence interval of aOR.

* *p* <.05, **p < .01, ***p < .001.

Table 4 furthermore shows that a large proportion of victims and nonvictims (between 44.2% and 75.5%) with partner/family, financial, and legal problems, and lack of support had any pre-existing mental health problem. Only with respect to lack of emotional support, victims significantly more often had any pre-existing mental health problems than nonvictims (68.5% vs. 46.2%).

A large proportion (between 79% and 97%) of both groups with current specific social/financial/legal problems already had any of these problems at T1/T2 (pre-existing problems) without significant differences between both groups. Only victims with a current lack of emotional support significantly more often had social/financial/legal problems in the past two years than nonvictims (87.5% vs. 79.8%).

### Incidence of (new) problems among victims and nonvictims

Table 3 additionally presents the incidence of (new) problems. Clearly, the incidence of problems is much lower than their prevalence. Only the incidence of general mental health problems was significantly higher among victims compared to nonvictims.

### Robustness of findings

A subsample of nonvictims was exposed to VAT in the 12 months before T1 and T2 (see Table 1). This could explain the absence of differences between victims and nonvictims in the substantial prevalence of problems before T3 among those with current problems at T3 and the substantial prevalence of any pre-existing mental health problem among those with current at T3. To rule out this possibility, we repeated the multivariate logistic regression analyses after excluding this subsample from the nonvictims group (*N* = 3,357). Results showed that excluding this subsample from the nonvictims group hardly affected the findings (see appendix 2). Only for “problems with partner/family” results differed from previous analysis (see Table 4): now, victims, more often had any pre-existing mental health problem among those with current problems with partner/family than nonvictims (73.9% vs. 48.1%, aOR = 2.95, 95% CI = 1.34–6.48, *p* = .007).

## Discussion

The presented findings allow for a straight-forward answer of the four main research questions:

(1) A substantial minority of victims as well as nonvictims suffered from (pre-existing) mental health, social, financial, and legal problems at T1 and/or T2. Prevalence of pre-existing problems in all domains is significantly higher in victims compared to nonvictims. (2) The same pattern emerges for current mental health, social, financial or legal problems at T3. (3) The central finding of the present study is that the large majority of adults recently exposed to potentially traumatic events (PTEs), for example, violence, accidents, and/or serious threats, with current post-event moderate-severe anxiety and depression symptomatology, general mental health problems, problems with partner/family, financial problems, and lack of emotional support, already had similar problems in the 2 years before the PTEs. With respect to a probable diagnosis PTSD, 85% of the victims with probable PTSD at T3 already suffered from moderate-severe ADS, probable PTSD, and/or GMHP in the 2 years before (at T1 and/or T2). Importantly, the results furthermore showed that victims and nonvictims hardly differed in the prevalence of pre-existing problems among those with current problems. Importantly, excluding nonvictims who were exposed to VAT in the past, hardly affected the results. (4) The incidence of (new) current problems was much lower than the prevalence of current problems. With the exception of general mental health problems (6.5% vs. 2.7% in victims and nonvictims, respectively), the incidence of current problems was not elevated in victims compared to nonvictims.

Our central finding that the vast majority of victims exposed to PTEs with current post-event problems already had similar problems in the 2 years before expands the results and conclusions of DiGangi et al. (2013). It demonstrates that many variables, previously considered outcomes of trauma, are not simply partly present before the potentially traumatic event, but are present among *the large majority* of victims with problems. Our results are also in line with a recent large case-control study reporting pre-trauma psychiatric diagnoses were the strongest predictor of severe post-trauma psychiatric comorbidity defined as ⩾3 psychiatric diagnosis during 5-year post-trauma interval (Gradus et al., 2022). Moreover, the findings of DiGangi et al. (2013), Danese et al. (2017), Scheeringa (2021), Gradus et al. (2022), and current findings question if some outcomes of the well-known meta-analysis of Brewin et al. (2000) and Ozer et al. (2003) on (bi-variate) risk factors for PTSD and PTSD symptom-severity are still valid. Both meta-analysis suffered from “heavy reliance on [. . .] retrospective designs” (Ozer et al., 2003, p. 67) which may be responsible for the outcome that the highest effect sizes were observed for peri- and post-traumatic factors rather than for pre-trauma mental health problems. Unfortunately, studies not equipped to assess the impact of pre-trauma variables without the bias of retrospective assessments (e.g. Rosellini et al., 2018) tend to repeat the common – but not empirically supported by prospective research – assumption that pre-trauma factors are of reduced significance when it comes to the effects of PTEs.

Interestingly, the above observed patterns with respect to pre-existing problems were – by the exception of lack of emotional support – almost similar among victims and nonvictims. These outcomes suggest that, when victims and nonvictims suffer from a specific problem (besides PTSD) and seek help, professional care providers may expect pre-existing problems among victims as often as among nonvictims.

Our central finding that the majority of problems were already present at T1 and/or T2 indicates that these problems may be best understood as pre-existing (persistent or recurrent) rather than as post-event problems. This may be counter-intuitive even for professional care providers, as PTSD is typically presented as a mental health problem resulting from exposure to specific traumatic events. For instance, on the site of the National Center for PTSD (https://www.ptsd.va.gov/), people may read “*PTSD is a mental health problem that some people develop after experiencing or witnessing a life-threatening event [. . .]”* suggesting that PTSD is primarily, if not exclusively, caused by the event. In this perspective, it is not strange that other post-event problems are viewed in a similar way: that these problems are primarily, if not exclusively, caused by these events (rather than as already being present before the event). The presented findings from this large prospective study show the opposite.

It needs, however, to be emphasized that mental health and other problems causing distress and impairment require adequate treatment, independently of understanding them as pre- or post-event problems. Moreover, some individuals without pre-existing mental health and other problems develop new problems after experiencing a PTE. In most cases, however, seemingly post-event problems appear to be typically a continuation of pre-event problems. PTEs may confirm pre-existing experiences, hinder recovery from pre-existing problems, or cause relapse, as it is known for depressive episodes (Kendler et al., 1995). In addition, when it is difficult to understand the origin of pre-event problems or if these are accompanied by experiences of stigmatization, PTEs may subsequently serve as an explanation or justification of their existence. In sum, post-event problems appear to be an expression of pre-event problems rather than new problems.

The finding that many post-event mental health, social, financial, and legal problems were in fact present before the PTE’s, may also help to explain the poor outcomes and drop-out rate of evidence-based PTSD treatments (Bradley et al., 2005; Lewis et al., 2020; Springer et al., 2018) typically focusing on symptoms considered as originating from a specific event. Chronic and complex problems are more difficult to treat (Gerger et al., 2014), and lower treatment gains may lead to higher dropout rates (Berke et al., 2019).

## Strengths and limitations

Major strengths of the present prospective study are the use of a large probability sample of the general adult population with high response rates and weighted data to optimize the representativeness of the study findings, the prospective study-design enabling the use of non-retrospective data on pre-existing problems, the inclusion of a comparison group of nonvictims, and multivariate analyses controlling for relevant confounders such as experiences of other PTEs/SLEs, and demographics.

Although we used validated questionnaires on mental health, we did not conduct clinical interviews to examine mental disorders. General mental health problems, problems in the areas of work, family/partnership, finances, as well as legal problems were assessed by each one item to reduce the burden of longer questionnaires. One-item measures may be less sensitive to detect differences between groups, although this and previous studies using the applied measure showed the opposite (van der Velden et al., 2019). Due to cell counts, we were not able to examine the differences between victims of violence, accidents, and serious threats. Our findings may not be applicable to extreme traumatic events such as torture, repeated (type-II; Terr, 1991) events (e.g. during a war), and adverse childhood experiences.

Although we simultaneously examined, in contrast to other studies among victims, a broad range of different problems victims and nonvictims may face, there are several other relevant markers of pre-trauma factors such as coping self-efficacy (Mahoney et al., 2019), personality (Fletcher et al., 2016), and parenting (Christie et al., 2019) for which future prospective studies among the general population with non-retrospective pre-event measures are required.

## Supplemental Material

sj-docx-1-isp-10.1177_00207640221140287 – Supplemental material for To what extent do post-traumatic mental health and other problems reflect pre-existing problems? Findings from the prospective comparative population-based VICTIMS-study Click here for additional data file. Supplemental material, sj-docx-1-isp-10.1177_00207640221140287 for To what extent do post-traumatic mental health and other problems reflect pre-existing problems? Findings from the prospective comparative population-based VICTIMS-study by Peter G van der Velden, Carlo Contino, Marcel Das and Lutz Wittmann in International Journal of Social Psychiatry
