# Supplementary material for: To what extent do post-traumatic mental health and other problems reflect pre-existing problems? Findings from the prospective comparative population-based VICTIMS-study
Source: Int J Soc Psychiatry. 2022 Dec 4;69(4):841–52. doi: 10.1177/00207640221140287 (PMC10248301; doi:10.1177/00207640221140287)
Supplement: sj-docx-1-isp-10.1177_00207640221140287 – Supplemental material for To what extent do post-traumatic mental health and other problems reflect pre-existing problems? Findings from the prospective comparative population-based VICTIMS-study [file sj-docx-1-isp-10.1177_00207640221140287.docx]

**Appendix 1**

|  | Total Exposure to VAT | Index-even VAT^1^ |
| --- | --- | --- |
|  | N (%) | N (%) |
| serious threat | 83 (24.4) | 59 (17.4) |
| online serious threat | 27 (7.9) | 10 (2.9) |
| sexual violence/abuse | 22 (6.5) | 14 (4.1) |
| online sexual violence/abuse | 13 (3.8) | 4 (1.2) |
| robbery | 15 (4.4) | 9 (2.6) |
| traffic accident | 121 (35.6) | 105 (30.9) |
| airplane accident | 4 (1.2) | 1 (0.3) |
| accident at work | 36 (10.6) | 24 (7.1) |
| fire | 33 (9.7) | 26 (7.6) |
| physical violence (not by own partner) | 22 (6.5) | 15 (4.4) |
| physical violence by own partner | 23 (6.8) | 12 (3.5) |
| medical error/accident | 71 (20.9) | 56 (16.5) |
| other violence | 7 (2.1) | 6 (1.8) |
|  |  |  |
| total | 477 | 341 |

^1^ Event victims took in mind when filling in the PCL5.

Due to weighting numbers may slightly differ.

**Appendix 2**

Victims (N=340) and nonvictims not victimized in 12 months before T1, T2 and T3 (N=3357).

|  |  | Prevalence problems before T3 among those with problems at T3^1^ | | | | Prevalence of any pre-existing mental health problem among those with current problems^2^ | | |
| --- | --- | --- | --- | --- | --- | --- | --- | --- |
|  | N^total^ | n (%) | aOR (95% CI) | | N^total^ | n (%) | aOR (95% CI) | |
| Moderate-severe anxiety and depression symptoms | | | | | |  |  |  |
| - nonvictims | 3357 | 328 (65.3) | 1 |  | 501 | 344 (68.7) | 1 |  |
| - victims | 340 | 70 (73.7) | 1.38 (0.83-2.29) | | 95 | 76 (80.0) | 1.71 (0.97-2.99) | |
| Posttraumatic stress-symptoms (VAT-related) | | | | |  |  |  |  |
| - nonvictims | 3357 |  |  |  |  |  |  |  |
| - victims | 341 | 32 (47.8) | n.a. |  | 67 | 58 (86.6) | n.a. |  |
| General mental health problems (GMHP) | | | | |  |  |  |  |
| - nonvictims | 3357 | 229 (71.1) | 1 |  | 322 | 266 (82.6) | 1 |  |
| - victims | 341 | 53 (70.7) | 0.85 (0.47-1.53) | | 74 | 59 (79.7) | 0.77 (0.40-1.49) | |
| Problems at work^3^ | |  |  |  |  |  |  |  |
| - nonvictims | 1724 | 51 (45.9) | 1 |  | 111 | 49 (44.1) | 1 |  |
| - victims | 179 | 14 (53.8) | 1.38 (0.59-3.24) | | 26 | 13 (50.0) | 1.20 (0.51-2.80) | |
| Problems with partner / family | | | |  |  |  |  |  |
| - nonvictims | 3357 | 120 (56.3) | 1 |  | 214 | 103 (48.1) | 1 |  |
| - victims | 340 | 31 (67.4) | 1.64 (0.83-3.22) | | 46 | 34 (73.9) | 2.95 (1.34-6.48)^**^ | |
| Financial problems | |  |  |  |  |  |  |  |
| - nonvictims | 3357 | 111 (62.7) | 1 |  | 177 | 85 (48.0) | 1 |  |
| - victims | 340 | 45 (76.3) | 1.80 (0.91-3.55) | | 59 | 38 (64.4) | 1.74 (0.91-3.33) | |
| Legal problems | |  |  |  |  |  |  |  |
| - nonvictims | 3357 | 16 (41.0) |  |  | 39 | 13 (33.3) | 1 |  |
| - victims | 340 | 11 (57.9) | n.c. |  | 19 | 10 (52.6) | 1.65 (0.45-6.05) | |
| Lack of emotional support | | |  |  |  |  |  |  |
| - nonvictims | 3357 | 533 (71.4) | 1 |  | 746 | 322 (43.2) | 1 |  |
| - victims | 340 | 95 (79.2) | 1.46 (0.90-2.37) | | 120 | 80 (66.7) | 2.28 (1.50-3.48^)***^ | |

^1^ Problems at T1 and/or T2 among those with corresponding problems at T3 [percentage = (n^prevalence before T3 among T3^/n^prevalence T3^)^*^100.

^2^ Moderate-severe anxiety and depression symptomatology (ADS) and/or general mental health problems (GMHP) at T1 and/or T2.

^3^Among those employed at T3.

Due to weighting numbers may slightly differ.
